# Tractable targets for meropenem-sparing antimicrobial stewardship interventions

**DOI:** 10.1093/jacamr/dlz042

**Published:** 2019-09-06

**Authors:** Clark D Russell, Ian F Laurenson, Morgan H Evans, Claire L Mackintosh

**Affiliations:** 1 Regional Infectious Diseases Unit, NHS Lothian Infection Service, Western General Hospital, Edinburgh, UK; 2 University of Edinburgh Centre for Inflammation Research, Queen’s Medical Research Institute, Edinburgh BioQuarter, Edinburgh, UK; 3 Clinical Microbiology, NHS Lothian Infection Service, Laboratory Medicine, Royal Infirmary of Edinburgh, Edinburgh, UK

## Abstract

**Background:**

As meropenem is a restricted antimicrobial, lessons learned from its real-life usage will be applicable to antimicrobial stewardship (AMS) more generally.

**Objectives:**

To retrospectively evaluate meropenem usage at our institution to identify targets for AMS interventions.

**Methods:**

Patients receiving meropenem documented with an ‘alert antimicrobial’ form at two tertiary care UK hospitals were identified retrospectively. Clinical records and microbiology results were reviewed.

**Results:**

A total of 107 adult inpatients receiving meropenem were identified. This was first-line in 47% and escalation therapy in 53%. Source control was required in 28% of cases after escalation, for predictable reasons. Those ultimately requiring source control had received more prior antimicrobial agents than those who did not (*P* = 0.03). Meropenem was rationalized in 24% of cases (after median 4 days). Positive microbiology enabled rationalization (OR 12.3, 95% CI 2.7–55.5, *P* = 0.001) but rates of appropriate sampling varied. In cases with positive microbiology where meropenem was not rationalized, continuation was retrospectively considered clinically and microbiologically necessary in 8/40 cases (0/17 empirical first-line usage). Rationalization was more likely when meropenem susceptibility was not released on the microbiology report (OR 5.2, 95% CI 1.3–20.2, *P* = 0.02). Input from an infection specialist was associated with a reduced duration of meropenem therapy (*P* < 0.0001). Early review by an infection specialist has the potential to further facilitate rationalization.

**Conclusions:**

In real-life clinical practice, core aspects of infection management remain tractable targets for AMS interventions: microbiological sampling, source control and infection specialist input. Further targets include supporting rationalization to less familiar carbapenem-sparing antimicrobials, restricting first-line meropenem usage and selectively reporting meropenem susceptibility.

## Introduction

Careful antimicrobial stewardship (AMS) is essential to decelerate the emergence of resistance to currently available drugs, and thus preserve their efficacy. Trials of AMS interventions demonstrate efficacy in reducing rates of both infection and colonization with antimicrobial-resistant (AMR) bacteria.^1^^,^^2^ Importantly, the associated reduction in inpatient antimicrobial usage has not been associated with an increase in mortality.

In comparison with other β-lactams, resistance to carbapenems is less prevalent; thus their broad spectrum of antimicrobial activity remains relatively preserved. However, the prevalence of carbapenem resistance throughout the world is increasing and usage of carbapenems should be restricted, unless absolutely necessary, to maintain their efficacy.^3–7^ Recognizing this, carbapenems are considered ‘critically important’ antimicrobials by the WHO. Carbapenem sparing, when clinically and microbiologically appropriate, is therefore a key goal of AMS programmes.

Meropenem is a restricted antimicrobial at our institution and should be reserved for life-threatening infections or AMR bacteria (e.g. ESBL-producing Gram-negative organisms). Meropenem usage should therefore exemplify best practice in infection management and lessons learned from critically evaluating real-life meropenem usage will be applicable to AMS more generally.

The Scottish Antimicrobial Prescribing Group (SAPG) recently published an evaluation of their guidance related to carbapenem usage, identifying good compliance with local prescribing policies, lack of confidence in de-escalating therapy and decreased usage during the 2012–16 study period.^8^ The aim of this study was to retrospectively evaluate cases of meropenem usage at our institution in depth to identify targets for AMS interventions. These data should complement the positive results from national quality improvement programmes (QIPs) such as the SAPG guidance by refining knowledge of the specific elements of clinical practice amenable to improvement through targeted QIPs.

## Methods

### Data collection

Meropenem is designated an ‘alert antimicrobial’ at our institution, meaning that it can be used after approval has been granted by an infection specialist (Medical Microbiologist or Infectious Diseases physician) or for limited pre-approved empirical indications (Table S1, available as Supplementary data at *JAC-AMR* Online) without the requirement for prior discussion with an infection specialist. An alert form should be completed for any meropenem prescription and this form is required for the hospital pharmacy to provide a supply of meropenem for any patient. Doripenem and imipenem are not routinely available and ertapenem is reserved for use in the Outpatient Parenteral Antimicrobial Therapy service. Alert forms received between July and October 2017 were retrospectively reviewed to identify patients receiving meropenem at our institution (two tertiary care hospitals in Edinburgh, UK, with a combined total of 1405 beds), which provides regional neurosurgery, oncology, haematology, cardiothoracic surgery, transplant (liver, kidney, pancreas and islet cell) and infectious diseases services. Diagnostic microbiology services are provided to both hospitals by the same laboratory. Clinical and microbiological data were gathered from the forms, electronic patient records and the microbiology laboratory information management system. Statistical analysis was performed using GraphPad Prism 8 for Mac OS, version 8.0.1.

### Assessing meropenem necessity

In a subset of cases where meropenem therapy was not rationalized and positive microbiology results were available, the necessity of continued meropenem usage was assessed retrospectively by two infection consultants. This assessment was based on the recorded reason for usage, infection diagnosis, sample type from which the organism was grown, antibiograms of organisms, antimicrobials already received for the infection, allergies and the outcome of any bedside reviews by infection specialists. Assessments were conducted independently with discussion with a third reviewer in cases of disagreement. On the basis of the recently reported MERINO trial, meropenem was considered preferable to piperacillin/tazobactam for treatment of infection with an ESBL-producing Gram-negative organism.^9^

### Statistics

Normal data distribution was tested for using the Shapiro–Wilk test. The Mann–Whitney test was used to compare groups not normally distributed and the *t*-test was used to compare normally distributed groups. Categorical variables were analysed using Fisher’s exact test. Statistical analyses were performed using GraphPad Prism version 7.0.

### Ethics

This project was conducted as part of ongoing AMS quality improvement, utilizing routinely collected data in accordance with the Caldicott principles, so further ethics approval was not required.

## Results

### Patient and infection characteristics

Alert forms for meropenem were received for 107 adult inpatients during the study period. The median patient age was 63 years (IQR 49.5–71.5) and 48% were female. Eighty-two percent of patients survived to hospital discharge. Most infections were hospital acquired (non-ICU setting 23%, ICU acquired 8%) or healthcare associated (37%, defined per Friedman *et al.*^10^), though 32% were community acquired. Meropenem was used most often by respiratory (25%), haematology/oncology (18%), critical care (12%) and neurosurgery (11%) services. The most common clinical diagnoses (Table 1) were respiratory tract infection (37%), urinary tract infection (13%), intra-abdominal infection (11%), neutropenic sepsis (8%) and post-neurosurgical CNS infection (6%).

**Table 1.** Clinical diagnoses of patients receiving meropenem

Diagnosis	*N* (%)^a^
Respiratory tract infection	41 (37.3)
bronchiectasis exacerbation	19 (17.3)
ventilator-associated pneumonia	9 (8.2)
hospital-acquired pneumonia	5 (4.5)
community-acquired pneumonia	4 (3.6)
cystic fibrosis exacerbation	4 (3.6)
Urinary tract infection	14 (12.7)
Intra-abdominal infection	12 (10.9)
Neutropenic sepsis	9 (8.2)
Post-neurosurgical CNS infection	6 (5.5)
Non-infectious diagnosis^b^	6 (5.5)
Other infection^c^	8 (7.3)
CNS infection	4 (3.6)
Surgical site infection	4 (3.6)
Bacteraemia, unknown source	2 (1.8)
Osteomyelitis	2 (1.8)
Skin and soft tissue infection	2 (1.8)

a *n* = 110 diagnoses as some patients had >1 diagnosis.

b Methotrexate pneumonitis (*n* = 1); ischaemic bowel (*n* = 1); fever due to cancer (*n* = 3); and unknown but infection not proven (*n* = 1).

c Line infection (*n* = 5); sepsis from unknown source (*n* = 1); infected spinal metalwork (*n* = 1); empyema and sub-phrenic collections (*n* = 1).

### Meropenem usage, prior antimicrobials and microbiological sampling

**Figure 1.**
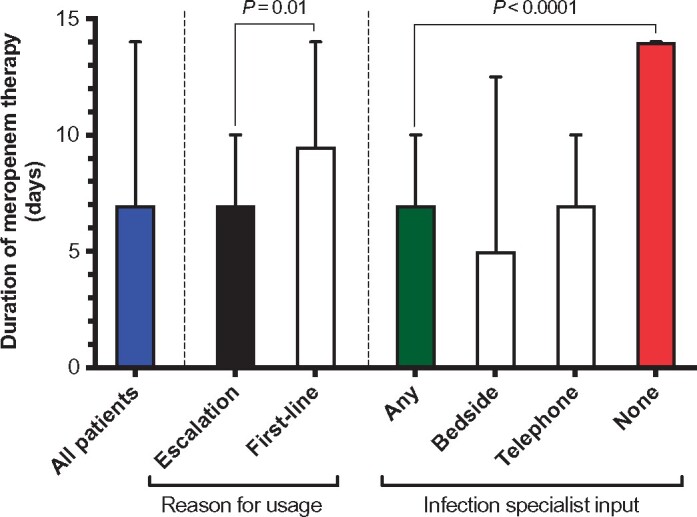
Duration of meropenem therapy expressed as median and IQR. Duration in indicated groups was compared using the Mann–Whitney test.

Meropenem was used as escalation therapy in 53% of cases and first-line in 47%. In cases of first-line usage this was empirical in 86% and in response to a microbiology result from a previous episode in 14%. Escalation of therapy to meropenem occurred after a median of 4 days (IQR 2–7) and use of a median of two prior antimicrobial agents (IQR 2–3). The most commonly used antimicrobials prior to escalation to meropenem were gentamicin (*n* = 32), amoxicillin (*n* = 20), metronidazole (*n* = 17), piperacillin/tazobactam (*n* = 16), vancomycin (*n* = 13) and ceftriaxone (*n* = 8). Amongst all patients, the median duration of meropenem usage was 7 days (IQR 5–14; Figure 1). When used as escalation therapy the median duration was also 7 days (IQR 4–10) compared with 9.5 (IQR 7–14) when used first-line (*P* = 0.01). Microbiological sampling was performed in 94% of patients overall, with blood cultures obtained in 71%. Appropriate sampling was performed least frequently in patients with respiratory tract infections (Figure S1), which were the most common reason for meropenem usage.

### Requirement for source control after escalation to meropenem

An intervention to achieve source control was required in 16/57 (28%) cases after escalation to meropenem. These patients had received a median of three prior antimicrobials (compared with two for patients escalated who did not require source control, *P* = 0.03). The foci of infection and interventions required were usually predictable reasons for prior antimicrobials to have failed: serious intra-abdominal infections requiring surgery (*n* = 4), concern for line infection leading to line removal (*n* = 5), drainage of ascites or a collection (*n* = 5) or removal of infected prosthetic material (*n* = 2).

### Infection specialist input

**Figure 2.**
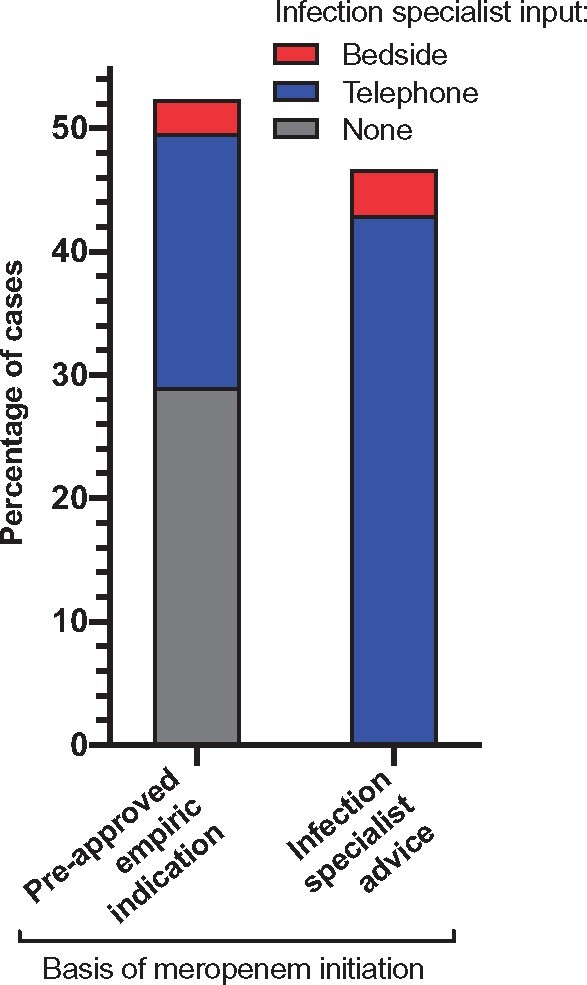
Infection specialist input leading to initiating meropenem therapy. The ‘infection specialist advice’ group denotes instances where meropenem was recommended by an infection specialist for an indication not included in the list of pre-approved empirical indications (Table S1).

Meropenem was used on the basis of a pre-approved empirical reason in 51% of cases (Table S1), in 29% with no telephone or bedside input from an infection specialist (Figure 2). The next most common scenario was meropenem initiation on the basis of telephone advice from an infection specialist (43%). In 6.5% of cases meropenem was prescribed following a bedside review by an infection specialist (including Infectious Diseases Unit inpatients).

In total, 21 patients had bedside infection specialist review (20%), including 4 who were managed in the Infectious Diseases Unit. Of the 17 patients reviewed at the bedside outside the Infectious Diseases Unit, meropenem was initiated on the basis of the visit in three cases (day 0) and in the remaining 14 the median time to review was 3 days (IQR 1–5). The outcome of the review was to stop (2/14) or switch meropenem to another antimicrobial (4/14) on the first review or switch on a subsequent review (2/14). Cumulatively in 8/14 cases the recommendation was not to continue meropenem (this advice was always followed by the patient’s primary team). In the remaining six cases, therapy had been rationalized prior to review in one, the patient was being palliated in one and continuing meropenem was recommended in four.

Overall, infection specialist input (telephone or bedside) was associated with a significantly shorter duration of meropenem therapy (median 7 versus 14 days, *P* < 0.0001, Figure 1). In cases where the outcome of a bedside review was to recommend stopping/switching meropenem the median duration of meropenem was 4 days (IQR 3–5) compared with 7 days in the entire cohort.

### Risk factors for MDR organisms in patients receiving meropenem

One or more risk factors for carriage of an MDR organism were present in the majority (78%) of patients (Table S2).^11–15^ Inadequate data were available to reliably comment on preceding international travel, therefore this was not included. At least 55% had received antimicrobials in the preceding year (including 14% receiving piperacillin/tazobactam), though this figure will be an underestimate as primary care prescription records were not reviewed. An ESBL-producing organism had been isolated from 25% of patients in the past.

### Microbiology results and rationalization of therapy

**Figure 3.**
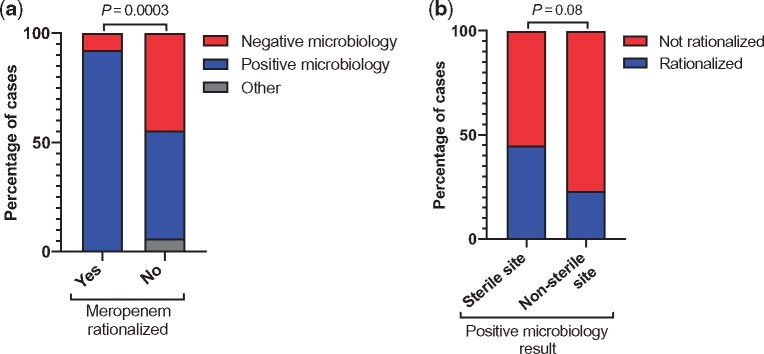
Influence of microbiology results on meropenem rationalization. Proportions were compared using Fisher’s exact test. (a) Positive and negative microbiology. (b) Rationalized and not rationalized. Other: patient died or inadequate data.

A positive microbiology result was obtained in 65% of patients (69/107), with growth from a sterile site (blood, CSF, intra-operative sample or aspirate of intra-abdominal, articular or pleural fluid) in 38 cases and a non-sterile site (sputum, bronchoalveolar lavage, tracheal aspirate, swab, urine) in 31 cases (Table S3). Meropenem was rationalized in a total of 26 cases (24%; after a median of 4 days, IQR 2–5) and continued in 74 cases. The patient died during therapy in six cases and inadequate data were available for one case. A positive microbiology result was strongly associated with rationalization of therapy (OR 12.3, 95% CI 2.7–55.5, *P* = 0.001; Figure 3a). Twenty-four of 26 instances of meropenem rationalization occurred in response to positive microbiology. A positive result from a sterile site (17/38) led to more rationalization than a result from a non-sterile site (7/31, *P* = 0.08; Figure 3b).

When meropenem susceptibility was not released on the microbiology report available to clinicians, rationalization to a carbapenem-sparing agent was more likely (OR 5.2, 95% CI 1.3–20.2, *P* = 0.02).

### Necessity of continued meropenem usage

Amongst the 74 cases where therapy was continued, positive microbiology results were available for 40 cases and amongst these continued meropenem use was retrospectively considered necessary in a total of 8 cases (20%; Table 2). In the remaining cases a narrower-spectrum alternative was considered microbiologically and clinically appropriate. Infection was due to an ESBL-producing Gram-negative bacterium in seven cases (bacteraemia *n* = 3, sputum *n* = 1, urine *n* = 3) but in four cases carbapenem-sparing agents (not piperacillin/tazobactam) were appropriate alternatives; therefore continued meropenem usage was not considered necessary. First-line meropenem usage was considered necessary in 1/19 cases for treatment of *Pseudomonas aeruginosa* pneumonia in a patient with a history of ESBL-producing organism isolation. Continued use was considered necessary in 0/17 cases where first-line meropenem was empirical, with no prior microbiology suggesting it would be required. Of the patients escalated to meropenem (21/40), source control was required in 1/7 cases where continued use was considered necessary and 6/14 where it was considered unnecessary. Considering the 32/40 cases where continued meropenem usage was not considered necessary, 355 days of meropenem therapy could theoretically have been avoided, quantified as 1065 DDD.

**Table 2.** Retrospective assessment of necessity of continued meropenem usage in cases with positive microbiology where therapy was not rationalized

Reason for meropenem usage	Positive microbiology	Meropenem rationalized	Meropenem continued	Continued meropenem usage necessary^a^
All patients	64	24	40	8/40
First-line therapy				
empirical	25	8	17	0/17
previous microbiology	5	3	2	1/2
Escalation therapy	34	13	21	7/21

Values are shown as numbers of patients.

a Retrospectively assessed by two infection consultants based on the recorded reason for usage, infection diagnosis, sample type from which organism was grown, antibiograms of organisms, antimicrobials already received for the infection, allergies and the outcome of any bedside reviews by infection specialists.

### Alternative antimicrobials for pathogen-directed therapy

**Figure 4.**
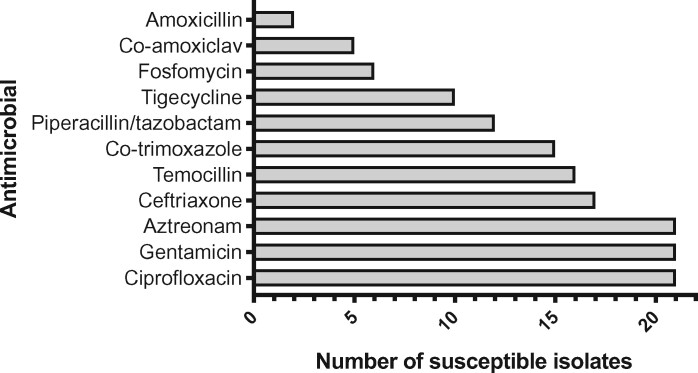
Rationalization options for pathogen-directed treatment of Gram-negative bacteria. Susceptibility tests for all listed antimicrobials were not performed for all isolates; therefore there is no consistent denominator for each antimicrobial. Results are shown for 35 bacterial isolates recovered from infections where meropenem therapy was continued and not rationalized following positive microbiology.

In cases with positive microbiology where meropenem was continued and not rationalized, Figure 4 shows the alternative antimicrobials that Gram-negative isolates were susceptible to. The β-lactams temocillin and aztreonam and non-β-lactam alternatives such as co-trimoxazole, gentamicin and ciprofloxacin were frequently active against bacteria identified.

## Discussion

The efficacy of AMS interventions and implementation is increasingly studied, with a recent Cochrane Library review published in 2017,^2^ and identifying specific elements of clinical practice to target with such interventions is valuable. This retrospective, in-depth evaluation of real-life meropenem usage has identified several tractable targets for carbapenem-sparing AMS interventions that could be incorporated into national AMS guidance, such as the recently evaluated SAPG QIP.^8^ These targets are likely to be relevant to antimicrobials other than carbapenems since carbapenem utilization should exemplify best practice in antimicrobial prescribing and infection management.

This study is limited by its retrospective nature and reliance on all relevant decision-making information being recorded in the electronic patient record. The parameters used to assess meropenem necessity represent an over-simplification of clinical decision making and therefore could underestimate the number of cases where meropenem usage was necessary for reasons other than those reviewed, but the decision to restrict this analysis to cases with positive microbiology results should ameliorate this limitation. The time period included in this review overlaps with a shortage of piperacillin/tazobactam, which may also have influenced some antimicrobial decisions, although in cases with positive microbiology available there were no organisms where the only rationalization option was piperacillin/tazobactam. Another limitation is an inability to identify and review potential cases where meropenem was used but no alert form was completed (though in such cases continued usage of meropenem should not be possible as an alert form is required for the pharmacy to release the drug).

Based on our data we have identified the following seven areas of practice as targets for carbapenem-sparing AMS interventions.

### 1. Microbiological sampling

The basic step of microbiological sampling, particularly prior to antimicrobial administration, is unsurprisingly critical to generate data to allow therapy to be rationalized and was strongly associated with rationalization of meropenem. Whilst the site of infection dictates which specific samples may be obtained, blood cultures are always possible but were not performed in 29% of cases.

### 2. Source control

Source control was required in 28% of cases after escalating therapy to meropenem (after receiving more prior antimicrobials than patients not requiring source control), and was usually a predictable contributor to antimicrobial failure (e.g. collections and infected prostheses). When antimicrobials are failing despite several rounds of escalation the necessity of source control should be considered at an earlier stage, before escalating to meropenem.

### 3. Infection specialist input

Infection specialist input, by bedside review or telephone consultation, was associated with a reduced duration of meropenem therapy. Overall, 20% of patients receiving meropenem were seen at the bedside by an infection specialist. Considering such cases (outside the Infectious Diseases Unit), the recommendation was to stop/switch meropenem in 8/17 cases. This was associated with a reduction in meropenem duration to a median of 4 days (versus 7 days for the whole cohort). In 9/17 cases the review happened >1 day after starting meropenem. We suggest that increased capacity for early, potentially unsolicited, bedside review of patients receiving meropenem has the potential to reduce duration of usage. Bedside review by an infection specialist is already known to improve AMS^16^ and also clinical outcomes in bacteraemia (including specifically *Staphylococcus aureus* bacteraemia), AMR bacterial infection and candidaemia.^17–20^

### 4. Approve limited duration of meropenem

Microbiological diagnosis of organisms susceptible to antimicrobials other than meropenem (Figure 4) often did not lead to rationalization of therapy. In cases where meropenem was rationalized this was done after a median of 4 days. Supply of a limited duration of meropenem (e.g. through provision of a permission code for a defined number of days) with the requirement for re-discussion with an infection specialist on day 4 could increase the proportion of cases where therapy is rationalized, rather than completing a 7 day course by default. A retrospective before-and-after study, comparing initial authorization of restricted antimicrobials with the additional requirement for re-authorization on day 3, reported reduced duration of usage of restricted antimicrobials and no difference in hospital mortality.^21^

### 5. Do not report meropenem susceptibility, where appropriate, when releasing microbiology results

In cases with positive microbiology, not reporting meropenem susceptibility was associated with increased likelihood of therapy being rationalized to a carbapenem-sparing agent. Therefore, the necessity of reporting meropenem susceptibility when releasing microbiology results should be carefully considered and avoided if the organism is susceptible to appropriate alternatives.

### 6. First-line meropenem usage

First-line meropenem use was common (47%), associated with an increased duration of therapy, and continuation was usually unnecessary in our retrospective assessment of cases with positive microbiology (18/19 unnecessary). In the one case where it was considered necessary, this was predicted by previous isolation of an ESBL-producing organism from the patient. Therefore, use of meropenem as a first-line antimicrobial in the absence of previous specific microbiology (prior ESBL organism isolation) is not supported by the results of this study and should be avoided. Carbapenems should be maintained as reserve antimicrobials (not in routine empirical recommendations/guidelines) with regular review of local resistance patterns and clinical use.

### 7. Increase familiarity with carbapenem-sparing antimicrobials

Apparent reluctance to use these alternatives to rationalize meropenem therapy could relate to non-infection specialist clinicians being unfamiliar with less commonly used antimicrobials (Figure 4). A recent meta-analysis identified a similar association between carbapenem usage and *Clostridioides* (*Clostridium*) *difficile* infection (CDI, OR 1.84) and quinolone usage (OR 1.66), providing some justification for quinolone usage as a carbapenem-sparing strategy without excess risk of CDI.^22^ Following publication of the MERINO trial it is important to note that in four out of seven cases where meropenem was continued for an ESBL-producing Gram-negative organism this was not necessary as other agents (not piperacillin/tazobactam) would have been suitable alternatives.

**Figure 5.**
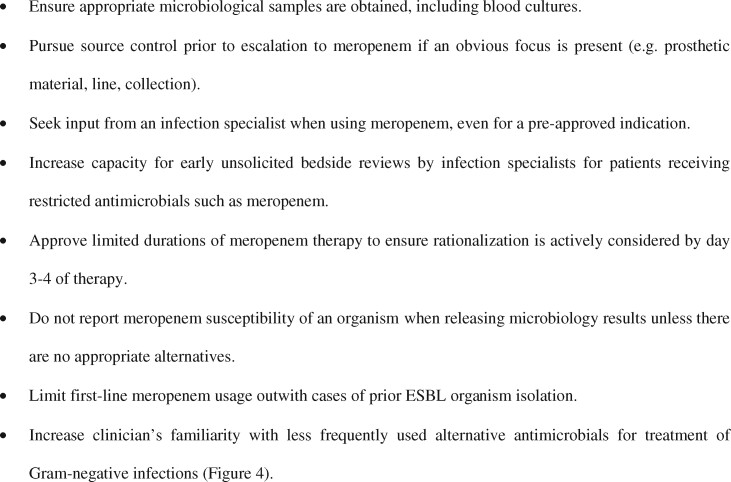
Tractable targets for meropenem-sparing AMS interventions.

By using carbapenem usage as a probe, we highlight that in real-life clinical practice core aspects of infection management represent simple, tractable targets for AMS interventions (Figure 5). The importance of these elements of practice should be continually emphasized to all clinicians and warrant inclusion in national QIPs. To derive the maximum benefit from molecular diagnostic technologies, and eventually novel therapeutics, it is critical to ensure ‘the basics’ of managing infection are consistently done well as a core element of AMS.

## Supplementary Material

dlz042_Supplementary_Data
