# Supplementary material for: Tractable targets for meropenem-sparing antimicrobial stewardship interventions
Source: JAC Antimicrob Resist. 2019 Sep 6;1(2):dlz042. doi: 10.1093/jacamr/dlz042 (PMC8210134; doi:10.1093/jacamr/dlz042)
Supplement: dlz042_Supplementary_Data [file dlz042_supplementary_data.zip › Supplementary_data.docx]

**Supplementary data**

**Figure S1: Microbiological sampling stratified by five most common infection sites**

**Table S1: Pre-authorised empiric indications for meropenem usage**

| From NHS Lothian Alert Antibiotic Policy for use in prescribing antibiotics for adults:   1. Febrile neutropenia, in accordance with haematology sepsis protocol 2. Second or third line agent for treatment of:    1. ventilator associated pneumonia    2. sepsis in the ITU setting 3. Treatment of neurosurgical patients as directed by microbiologist 4. Exacerbation of cystic fibrosis in accordance with the CF treatment algorithm 5. Exacerbation of bronchiectasis, in accordance with the treatment algorithm |
| --- |

**Table S2: Presence of MDRO risk factors in patients receiving meropenem**

| MDRO risk factor | N (%) |
| --- | --- |
| Antimicrobials in preceding year | 59 (55.1) |
| Piperacillin-tazobactam | 15 (14.0) |
| Vancomycin | 5 (4.7) |
| Previous ESBL-producing organism | 27 (25.2) |
| Immunosuppressed* | 26 (24.3) |
| Indwelling medical device | 25 (23.4) |
| Critical illness | 9 (8.4) |
| Number of risk factors present |  |
| 0 | 24 (22.4) |
| 1 | 43 (40.2) |
| 2 | 26 (24.3) |
| 3 | 13 (12.1) |
| 4 | 1 (0.9) |

*Immunosuppressive drug or haematological malignancy

**Table S3: Positive microbiology results**

| Organism | Sample type | N^a^ |
| --- | --- | --- |
| *Bacteroides uniformis* | Intra-abdominal aspirate | 1 |
| *Candida albicans* | Peripheral blood culture | 1 |
| *C. albicans* | Urine | 1 |
| *Candida glabrata* | Peripheral blood culture | 1 |
| *C. glabrata* | PICC tip | 1 |
| *Enterococcus avium* | Intra-abdominal aspirate | 1 |
| *Enterobacter cloacae* | Swab | 1 |
| *Escherichia coli* | Peripheral blood culture | 11 |
| *E. coli* | Urine | 11 |
| *E. coli* | Intra-abdominal aspirate | 5 |
| *E. coli* | BAL | 1 |
| *E. coli* | CSF | 1 |
| *E. coli* | Pleural aspirate | 1 |
| *E. coli* | Swab | 1 |
| *E. coli* | Tracheal aspirate | 1 |
| *Enterococcus faecalis* | Urine | 2 |
| *E. faecalis* | BAL | 1 |
| *E. faecalis* | Intra-abdominal aspirate | 1 |
| *E. faecalis* | Intra-operative | 1 |
| *E. faecalis* | Peripheral blood culture | 1 |
| *E. faecalis* | Swab | 2 |
| *Enterococcus faecium* | Intra-abdominal aspirate | 3 |
| *E. faecium* | Peripheral blood culture | 1 |
| *E. faecium* | Pleural aspirate | 1 |
| *Hafnia alvei* | BAL | 1 |
| *Haemophilus influenzae* | Sputum | 2 |
| *H. influenzae* | BAL | 1 |
| *Klebsiella oxytoca* | BAL | 2 |
| *K. oxytoca* | Peripheral blood culture | 2 |
| *Klebsiella pneumoniae* | BAL | 2 |
| *K. pneumoniae* | Peripheral blood culture | 2 |
| *K. pneumoniae* | Urine | 1 |
| *Micrococcus luteus* | Intra-operative | 1 |
| *Morganella morganii* | BAL | 1 |
| *Pseudomonas aeruginosa* | Sputum | 7 |
| *P. aeruginosa* | Peripheral blood culture | 3 |
| *P. aeruginosa* | Intra-abdominal aspirate | 1 |
| *Pasteurella spp.* | Peripheral blood culture | 1 |
| *Proteus spp.* | Sputum | 1 |
| *Rothia mucilaginosa* | Line blood culture | 1 |
| *Streptococcus anginosus* | Intra-abdominal aspirate | 1 |
| *Staphylococcus aureus* | Intra-operative | 3 |
| *S. aureus* | Sputum | 3 |
| *S. aureus* | BAL | 1 |
| *S. aureus* | Line blood culture | 1 |
| *S. aureus* | Swab | 1 |
| *Staphylococcus epidermidis* | CSF | 2 |
| *S epidermidis* | Intra-operative | 2 |
| *S. epidermidis* | Articular aspirate | 1 |
| *S. epidermidis* | Line blood culture | 1 |
| *Streptococcus gallolyticus* | Peripheral blood culture | 1 |
| *Staphylococcus haemolyticus* | Intra-operative | 1 |
| *Staphylococcus intermedius* | Sinus aspirate | 1 |
| *Serratia maracascens* | Sputum | 1 |
| *S. marcescens* | Intra-operative | 1 |
| *S. marcescens* | Sputum | 1 |
| *S. marcescens* | Tracheal secretions | 1 |
| *Streptococcus mitis* | Line blood culture | 2 |
| *Streptococcus oralis* | Intra-operative | 1 |
| *Streptococcus pneumoniae* | BAL | 1 |
| *S. pneumoniae* | CSF | 1 |
| *S. pneumoniae* | Intra-abdominal aspirate | 1 |
| *S. pneumoniae* | Peripheral blood culture | 1 |
| *Streptococcus salivarius* | Peripheral blood culture | 1 |

^a^ 112 organisms from 69 patients
