# Supplementary material for: Tractable targets for meropenem-sparing antimicrobial stewardship interventions
Source: JAC Antimicrob Resist. 2019 Sep 6;1(2):dlz042. doi: 10.1093/jacamr/dlz042 (PMC8210134; doi:10.1093/jacamr/dlz042)
Supplement: dlz042_Supplementary_Data [file dlz042_supplementary_data.zip › Reviewer reports.zip!/Reviewer report 2.docx]

**Reviewer report 2 (R0 – review of original submission)**

**PLEASE NOTE: JAC-AMR aims to publish your anonymous peer review comments with the final article for transparency. Please indicate in the box below whether you give your consent for us to publish your peer review comments for this article.**

Yes

**Synopsis**

**1. If the article contains original data, does the synopsis adequately reflect the manuscript as a whole, with the most important aspects described (of the introduction, methods and key results, and a balanced conclusion)? If NO, what is required to improve the synopsis? Please use the box below.**

No

Line 42: "more" may be misinterpreted by the abstract reader as meaning a greater number of days of antibiotics, but I do not think that is what the authors have reported. This requires clarification. 

With regard to the title, I would suggest using a more accessible word that "tractable," and also would recommend replacing "carbapenem" with "meropenem" as strictly speaking this was the only antibiotic that was assessed.

**Introduction**

**2. Does the introduction provide the necessary background information for readers to understand the article? (e.g. does it describe the problem, current state of knowledge, etc.) If NO, what is required to improve it? Please use the box below**

No

**3. Is the research hypothesis and/or aims/objectives of the study clearly stated in the introduction? If NO, what is required to improve it? Please use the box below**

Yes

**4. Is there superfluous/unnecessary text in the introduction? (Only text that is required for readers to understand why the study has been done should be included). If YES, what text is superfluous and should be removed? Please use the box below**

No

Considering the answers above and any other comments/advice you may have, please indicate what is required to improve the Introduction:

Line 63: I would suggest the authors avoid being so definitive with the idea that "reducing antimicrobial usage has not been associated with an increase in mortality" in light of the 2019 BMJ article Gharbi et al  https://doi.org/10.1136/bmj.l525. See also accompanying editorial http://press.psprings.co.uk/bmj/february/antibioticsedit.pdf.

References used on Line 69 are relatively old (both from 2011) and more up to date references should be cited to support the assertions made between lines 67-69

**Methods**

**5. Were the methods used suitably robust and appropriate for the research question(s) and/or the aims/objectives of the study? If NO, the article should be rejected, but please advise the authors accordingly in the free-text box below and complete the review in full**

Yes

**6. Are the methods described in enough detail so the reader can understand what was done and interpret the results and conclusions appropriately?**

Yes

**7. Are the methods described in language that a reader who does not specialize in the area of the study/methods used will understand? For example, are the statistical approaches used described in language that non-statistician healthcare professionals will understand? If NO, please indicate what is required to improve the language in the methods section in the box below**

No

Considering the above and any other comments/advice you may have, please indicate other aspects of the methods section that could be improved in the free-text box below)

The methods section requires a brief description of the statistical test(s) used, and, ideally, the rationale for them, Eg "categorical variables were analysed using ...... test"

The authors should describe how cases were categorised, if the two independent reviewers disagreed with regard to the necessity for ongoing meropenem use.

**Results**

**8. Given the methods used, are results described accurately, in enough detail and in an appropriate format to adequately inform the discussion section and the authors/readers conclusions? If NO, please indicate what is required to improve the results section in the box below**

No

**9. Based on what is described in the methods section, are data/results (that are likely to be available to the authors) missing from the current draft that you think should be presented to improve the manuscript?**

No

**10. Are Tables/Figures clear, required and do they add/complement the results text (they should not simply repeat what is in the text or vice versa)? If NO, please indicate what is required to improve the Tables/Figures in the box below**

No

Considering the above and any other comments/advice you may have, please indicate what is required to improve the results section:

A definition of ICU-acquired infection should be provided. I have presumed that the definitions of hospital-acquired and community-acquired infections are as per Friedman et al (reference 7), but this could be made clearer. 

Lines 139 and 154: Meropenem was used as escalation therapy in 54% of cases, which would be 58 cases out of the 107. However, the denominator used to describe cases "after escalation to meropenem" on line 154 was only 51. This apparent discrepancy should be explained. 

Line 142: With regard to the reporting of the number of antibiotics used prior to escalation, I would suggest clarifying this section to make it clear it is the number of different antibiotics given before escalation that is being reported, regardless of whether they were given in combination or sequentially. This will avoid this being interpreted as the number of antibiotic regimens used prior to escalation.

If the data are available, it would be interesting to know in how many cases meropenem was able to be rationalised following source control. 

Line 182: The reporting of a p value here seems inappropriate, as it would appear that a statistical test has been used to compare a subset of a cohort with the entire cohort, despite the fact that the same data is included in both groups. The discussion of this in Line 275 should similarly also be amended. 

Table 1: The total number of cases appears to add up to 110, whereas there were 107 cases as reported in the results section. Presumably some patients were designated as having more than 1 diagnosis, but this should be clarified underneath the table. One of the patients with a "Non-infectious diagnosis" was designated as having an "unknown" diagnosis. If the diagnosis was unknown, it is not clear to me how the authors concluded it was not an infection. This should be explained or that one case should be removed from that group. 

Table 3: I believe the use of the Mann Whitney test here is inappropriate, as what is being reported here is categorical data (eg. rationalised vs not rationalised). The "other" cases in Figure 3a, presumably refers to those who died or for whom inadequate information was available, but this should be clarified in the figure.

Given the p value reported, I would suggest the authors are not so definitive with their assertion in the sentence between lines 202-204.

**Discussion**

**11. Are the discussion and any conclusion(s) reasonable given the stated hypothesis/aims/objectives and results? If NO, please indicate how to improve the discussion in the box below**

Yes

**12. Is there superfluous/unnecessary text in the discussion? (Only text that directly relates to the results and understanding thereof in the context of existing published literature and the limitations of the study should be included.) If YES, please indicate what should be removed to improve the discussion in the box below**

No

**13. Is the Limitations sub-section adequate and reflective of the actual limitations of the study? ? If NO, please indicate how to improve the Limitations section in the box below**

Yes

Considering the above and any other comments/advice you may have, please indicate how to improve the discussion section:

As much of the unnecessary usage of meropenem appears to have occurred following pre-approved empiric indications (as described in supplementary table 1), the authors may wish to consider discussing whether further restriction of prescribing meropenem for these pre-approved indications is warranted.

**General**

**14. Is referencing throughout the manuscript satisfactory? (for instance are there statements for which you believe a supporting reference should be cited?) If NO, please indicate how to improve referencing in the box below**

Yes

**15. Is the language used (i.e. spelling, grammar, etc.) of high enough quality to be published as is? If NO, please indicate how to improve the language within the article in the box below**

Yes

**16. Are ethics/other approvals appropriate/adequate and satisfactorily described? ? If NO, please indicate how to improve this in the box below**

Yes

Considering the above and any other comments/advice you may have, please advise the authors how to improve these aspects of the article.

**Opinion and review articles only**

**17. Please advise the authors in the box below on how to improve their opinion or review article:**

|  **Recommendation** | |
| --- | --- |
|  | Accept (as is with no revisions) |
|  | Minor revision (with anticipation of subsequent acceptance if revisions are adequate) |
|  | Major Revision |
|  | Reject |

| **Would you be willing to review a revision of this manuscript?** | |
| --- | --- |
|  | Yes |
|  | No |

**Comments**

 Comments to the Author

If you have any additional comments to the authors about any aspect of the manuscript not covered in the structured review above, please place these in the box below. If you have no additional comments, please say “No additional comments” in box.

Overall, this is a well written manuscript which provides useful "real-world" data on meropenem prescribing, and the effects of a variety of antimicrobial stewardship interventions.
