# Supplementary material for: Tractable targets for meropenem-sparing antimicrobial stewardship interventions
Source: JAC Antimicrob Resist. 2019 Sep 6;1(2):dlz042. doi: 10.1093/jacamr/dlz042 (PMC8210134; doi:10.1093/jacamr/dlz042)
Supplement: dlz042_Supplementary_Data [file dlz042_supplementary_data.zip › Reviewer reports.zip!/Reviewer report 3.docx]

**Reviewer report 3 (R1 – review of revised submission)**

|  **Recommendation** | |
| --- | --- |
|  | Accept (as is with no revisions) |
|  | Minor revision (with anticipation of subsequent acceptance if revisions are adequate) |
|  | Major Revision |
|  | Reject |

| **Would you be willing to review a revision of this manuscript?** | |
| --- | --- |
|  | Yes |
|  | No |

**Comments**

 Comments to the Author

Comments from the initial review have been adequately addressed and I believe this has improved the manuscript.
